# Description of a new species of *Platynus* Bonelli from the Appalachian Mountains of eastern North America (Coleoptera, Carabidae)

**DOI:** 10.3897/zookeys.163.2295

**Published:** 2012-01-09

**Authors:** Yves Bousquet

**Affiliations:** 1 Agriculture and Agri-Food Canada, Ottawa, Ontario K1A 0C6

**Keywords:** *Platynus*, Carabidae, new species, identification key, barcoding

## Abstract

A new species of the genus *Platynus* Bonelli, *Platynus daviesi*, is described from specimens collected in the Appalachian Mountains. The species is structurally most similar to *Platynus parmarginatus* Hamilton but differs in having the coloration of the body dorsally darker on average, the elytra proportionally longer and wider, the vertex and disc of pronotum with well impressed microsculpture, the elytral interval 3 with four or five discal setae in most specimens, and the median lobe of aedeagus less curved overall. DNA barcoding was performed on several species of eastern North American *Platynus* species and *Platynus daviesi* was found to be genetically distinct from *Platynus parmarginatus*. A key to the 12 species of *Platynus* found east of the Mississippi River is provided.

## Introduction

The genus *Platynus* Bonelli is a large, inadequately understood, and probably polyphyletic group belonging to the tribe Platynini. In Mexico alone, the genus contains an estimated 300 species (Liebherr and Will 1996: 301). The North American (north of Mexico) *Platynus* are much less diversified and relatively well known. Lindroth (1966, as the *decentis* and *hypolithos* groups included in the genus *Agonum*) treated all species then known except those restricted to the southwest and *Platynus prognathus* Van Dyke, an aberrant species known only from the holotype collected on Saint Simon Island, in southeastern Georgia. Liebherr and Will (1996) described three new species and presented a key to all 22 species found in North America north of Mexico.

While curating the Nearctic carabids of the Canadian National Collection of Insects, I found that two species were mixed under the name *Platynus parmarginatus* Hamilton. The purpose of this paper is to describe the unnamed species.

## Material and methods

All the specimens reported in this study belong to the Canadian National Collection of Insects, Ottawa, Ontario (CNC), the Carnegie Museum of Natural History, Pittsburgh, Pennsylvania (CMNH), and the University of New Hampshire Collection, Durham, New Hampshire (NHDE). No attempt was made to locate further specimens in other collections.

The following measurements were taken on several specimens of the new species and *Platynus parmarginatus*: length of the head (LH) from the anterior edge of the clypeus at middle to an imaginary line between the posterior edges of the eyes; width of head (WH) across the eyes; length of pronotum (LP) along the midline; maximum width of pronotum (WP); length of elytra (LE) measured from the tip of the scutellum to the apex of the right elytron; and maximum width of elytra (WE). The standard body length [SBL = LH + LP + LE] was used to express the body length.

DNA extraction, PCR and COI sequencing followed standard protocols and primers at the Canadian Centre for DNA Barcoding (http://www.dnabarcoding.ca/pa/ge/research/protocols); all data are registered in the Barcode of Life Database (BOLD). The taxon ID-tree was produced by neighbour-joining analysis on Kimura 2-parameter distances, using the analytical tool in BOLD. Only sequences with lengths above 400 base pairs were used in the analysis. Genetic distances were estimated with MEGA 5.05 (http://www.megasoftware.net; accessed 20 October 2011).

### Platynus daviesi

Bousquet sp. n.

urn:lsid:zoobank.org:act:95C15DCE-1DC3-413D-88B8-6DC5ECC9DD3C

http://species-id.net/wiki/Platynus_daviesi

Figs 1 2 4 

#### Type material.

Holotype (♂) labelled: “virg. Shenandoah N.P. Powell Gap 13.VI.1982, 2300’ Bousquet & Davies / Holotype Platynus daviesi Bousquet CNC no 23464.” The specimen is deposited in the Canadian National Collection of Insects.

Paratypes (160♂♂, 127♀♀) from the following localities in the United States of America: Alabama. Natural Bridge Cave, Winston Co., 17.VI.61, H.R. Steeves [under rock, light zone] (1♂, CMNH). Monte Sano State Park, Hunstville, Madison Co., 6–8.IV.1991, R. Davidson, R. Acciavatti & M. Klingler (2♂♂, 5♀♀, CMNH). Connecticut. Burnham Brook, East Haddam, Middlesex Co., 16.VI.1980, 24.VII.1980, A.J. Main & W.L. Krinsky [malaise trap over brook] (2♀♀, CMNH). Kentucky. Foxtown, Jackson Co., 4.V.1984 (8♂♂, 11♀♀, CMNH). Carter Caves, Carter Co., 28.VII.1983, A. Larochelle (1♂, CMNH). Maryland. Garrett St. For., Garrett Co., 5.VI.96, J. Glaser (1♀, CMNH). New York. W. Shokan, Ulster Co., various dates, M.S. Adams [256 m, UV Light, Mesic forest hemlock] (9♂♂, 8♀♀, CMNH). Olivebridge, Ulster Co., various dates, M.S. Adams [118 m, UV Light] (7♂♂, 6♀♀, CMNH). “Plivebridge”, Ulster Co., 13–14.VII.1995, M.S. Adams (1♂, CMNH). Ithaca, Tompkins Co., 15.VII.1980, J.E. Rawlins (1♀, CMNH). North Carolina. Blue Ridge Parkway near Craven Gap, 940 m, Buncombe Co., 5.VI.1986, A. Smetana (1♂, CNC). Blue Ridge Parkway, Wolf Mountain outlook, 1680 m, 26.V.1986, A. Smetana (1♂, CNC). Highlands, 3800’, Macon Co., 8.VI.1957, W.J. Brown (1♀, CNC). Wayah Bald, 5500’, Macon Co., 6.VII.1952, H.& A. Howden (1♀, CNC). Pennsylvania. Cook State Forest, 1.2 mi N Cooksburg, Jefferson Co., 6.VI.1997, D. Chandler (3♂♂, 2♀♀, NHDE). Pittsburgh, 1.VII.1922 (1♂, 1♀, CMNH). Powdermill Nature Res., nr Rector, Westmoreland Co., 13.IX.58 (1♂, CMNH); idem, 26.IV.–2.V.1982, 16–20.V.1982, 26.IX.–1.X.1981, 30.V.–9.VI.1983, R. Davidson (4♂♂, 1♀, CMNH). 3 km NE Lower Burrell, Westmoreland Co., 19.VII.1946 (1♂, CMNH). 4.9 km S Ludlow, Pigeon Run, McKean Co., 15.VI.1994, 18.VII.1994, 24.V.1995, 22.VI.1995, J. Deeds or M. Ricke [560 m, UV Light Trap] (1♂, 5♀♀, CMNH). 5.4 km ENE Donaldson, Tionesta Scenic Area, McKean Co., 15.VI.1994, 22.VI.1995, M. Ricke or J. Deeds [565 m, UV Light Trap] (1♂, 1♀, CMNH). 4.2 km SSE Donaldson, Rock Run, Warren Co., 13.VII.1994, 9.VII.1994, 19.VII.1994, 5.VIII.1994, 19.VIII.1994, 24.V. 1995, 22.VI.1995, J. Deeds or M. Ricke [540 m, UV Light Trap] (2♂♂, 5♀♀, CMNH). 4.6 km ESE Donaldson, Tionesta Scenic Area, Warren Co., 15.VI.1994, M. Ricke (1♀, CMNH). 2.2 km NW Truemans, Warren Co., 15.VI.1994, 5.VIII.1994, M. Ricke (3♂♂, CMNH). 6.4 km S Irvine, Hedgehog Run, Warren Co., 1.VIII.1995, J. Deeds (1♀, CMNH). 6 km E Cobham, Warren Co., 10.VI.1995, C. Bier, J. Deeds & T. Schumann (1♂, CMNH). 7.7 km SSW Cherry Grove, Warren Co., 25.VII.1995, J. Deeds (1♂, CMNH). Black’s Run, Oakmont, Allegheny Co., 19.IV.1982, R. Davidson (1♀, CMNH). 1.6 km WSW Truemans, near mouth of Minister Creek, Forest Co., 15.VI.1994, M. Ricke [380 m, UV Light Trap] (1♂, CMNH). 8.7 km N Kellettville, Forest Co., 14.VIII.1995, J. Deeds (1♂, CMNH). 3.9 km WSW Pigeon, Penoke Run, Forest Co., 8.VIII.1996, J. Isaac [marsh with alders, UV light trap] (1♀, CMNH). 1.3 km SW Nansen, East Branch Spring Creek, 21.VIII.1996, J. Isaac [riparian, hemlocks] (1♂, CMNH). 5 km SSW West Finley, Enlow Fork Wheeling Creek, Washington Co., 16.V.1986, J.E. Rawlins (1♂, 1♀, CMNH). Lycoming Creek, 0.9 km NW Bodines, Lycoming Co., 14.VI.2001, B.J. Ray & S.E. Hamsher (1♀, CMNH). 3.5 km NE Shanksville, Somerset Co., 13.V.1995, W.A. Zanol (1♀, CMNH). Hawk Mountain Sanctuary, 2.3 km W Eckville, Berks Co., 8.VII.1997, M. Monroe & M. Medina (1♂, CMNH). Hawk Mountain Sanctuary, 1.1 km WNW Eckville, 15.V.1998, 28.V.1998, 26.VI.1998, 13.VIII.1998, 14.IX.1998, various collectors (5♂♂, 3♀♀, CMNH). Gravel Lick, Clarion Co., 3.V.1994, W.A. Zanol (1♀, CMNH). Tennessee. Chimney Tops, Great Smoky Mountains National Park, 8 km S Gatlinburg, 8.VI.1982, Bousquet & Davies (1♂, 2♀♀, CNC). Smoky Mtn. Nat. Pk., Elkmont, 12.VII.1974, R.D. Ward (1♀, CMNH). Foster Falls, 10 km SE Tracy City, Marion Co., 31.V.1991, R. Davidson, W. Zanol & R. Acciavatti (3♂♂, 5♀♀, CMNH). Virginia. Powell Gap, Shenandoah Nat. Park, 2300’, 13.VI.1982, Bousquet & Davies (5♂♂, 2♀♀, CNC). Simmons Gap, Shenandoah Nat. Park, 2250’, 14.VI.1982, Bousquet & Davies (1♂, 1♀, CNC). McCormick Gap, Shenandoah Nat. Park, 2430’, 14.VI.1982, Bousquet & Davies (3♀♀, CNC). Skyline Drive, Shenandoah Nat. Park, 3140’, 18.VII.1976, R.D. Ward (1♀, CMNH). Compton Gap, Shenandoah Nat. Park, 17.VI.1981, R. Davidson (1♀, CMNH). Mountain Lake Biological Station, 3820’, 12 km E Pembroke [Giles Co.], 11.VI.1982, Bousquet & Davies (2♂♂, CNC). “Matthews Arm”, Rappahannock Co., 19.VII.1980, A. Larochelle (1♀, CNC). Elkton, Rockingham Co., 21.VII.1980, A. Larochelle (1♀, CNC). Hightown, Highland Co., 4.VII.1980, A. Larochelle (1♂, CMNH). Loft Mtn., Greene Co., 3.VII.1980, A. Larochelle (1♀, CMNH). “Troutdale”, Grayson Co., 7.VII.1980, A. Larochelle (1♂, CMNH). 3 mi W Dungannon, Scott Co., 15.VI.–15.VII.1994, E. van den Berghe (3♂♂, 1♀, CMNH). Cumberland Gap Nat. Park, Lee Co., 5.VII.1984, E. Censky (7♂♂, 4♀♀, CMNH). West Virginia. Harpers Ferry, Jefferson Co., 18.VI.1974, P. Van Buskirk (3♂♂, CNC). Hungry Beech Preserve, 1 km E Kettle, Roane Co., 5–8.VI.1995, Harrity, Davidson & Onore [deciduous forest] (9♂♂, 7♀♀, CMNH). Ice Mountain Preserve, 0.5 km E North River Mills, Hampshire Co., 14–17.VI.1995, 19–22.VII.1995, 20–22.IX.1995, various collectors [deciduous forest, light trap] (27♂♂, 17♀♀, CMNH). Slaty Mountain Preserve, 4 km NW Sweet Springs, Monroe Co., 2–5.VI.1995, 25–28.VII.1995, various collectors [shale barrens, light trap] (8♂♂, 6♀♀, CMNH). Fayette Station, Fayette Co., 1–2.VII.1990, 2–12.VII.1990, Acciavatti & Davidson (2♂♂, 1♀, CMNH). Burner Mt., 3 km N Bartow, Pocahontas Co., 6.VIII.1986, R.E. Acciavatti [ex tree trunk] (1♂, CMNH). North Fork Mountain, 6.3 or 6.5 km SSE Hopeville, Grant Co., 25.IV.1994, 31.V.1994, 7.VI.1994, D. Mitchell & L. Mennell (3♂♂, 3♀♀, CMNH). 3.7 km WNW Hopeville, Grant Co., 31.V.1994, 7.VI.1994, D. Mitchell & L. Mennell [oak/maple forest] (8♂♂, 1♀, CMNH). 9.8 km N Upper Tract, Pendleton Co., 25.V.1994, 7.VI.1994, 9.VI.1994, D. Mitchell & L. Mennell (7♂♂, 2♀♀, CMNH). U.S. 50 at Cheat R., Preston Co., 12.V.1983, R.E. Acciavatti (1♀, CMNH). 3.2 km NNE Bowden, Randolph Co., 11–19.V.1995, 19–21.VI.1995, 22–29.VI.1995, 1–6.VII.1995, 2.VIII.1995, 22–31.VIII.1995, L. Mennell (5♂♂, 2♀♀, CMNH). 3.7 km NNW Bowden, Randolph Co., 2.VIII.1995, 14.VIII.1995, L. Mennell (1♂, 1♀, CMNH). Near Alderson [Greenbrier Co.], 30.VI–1.VII.1936, G.M. Kutchka (1♂, CMNH). Falls of Mills Creek, 11 mi W Mill Point, Greenbrier Co., 2–8.V.1982, R. Davidson (1♂, CMNH).

#### Description.

**Figure 1.**
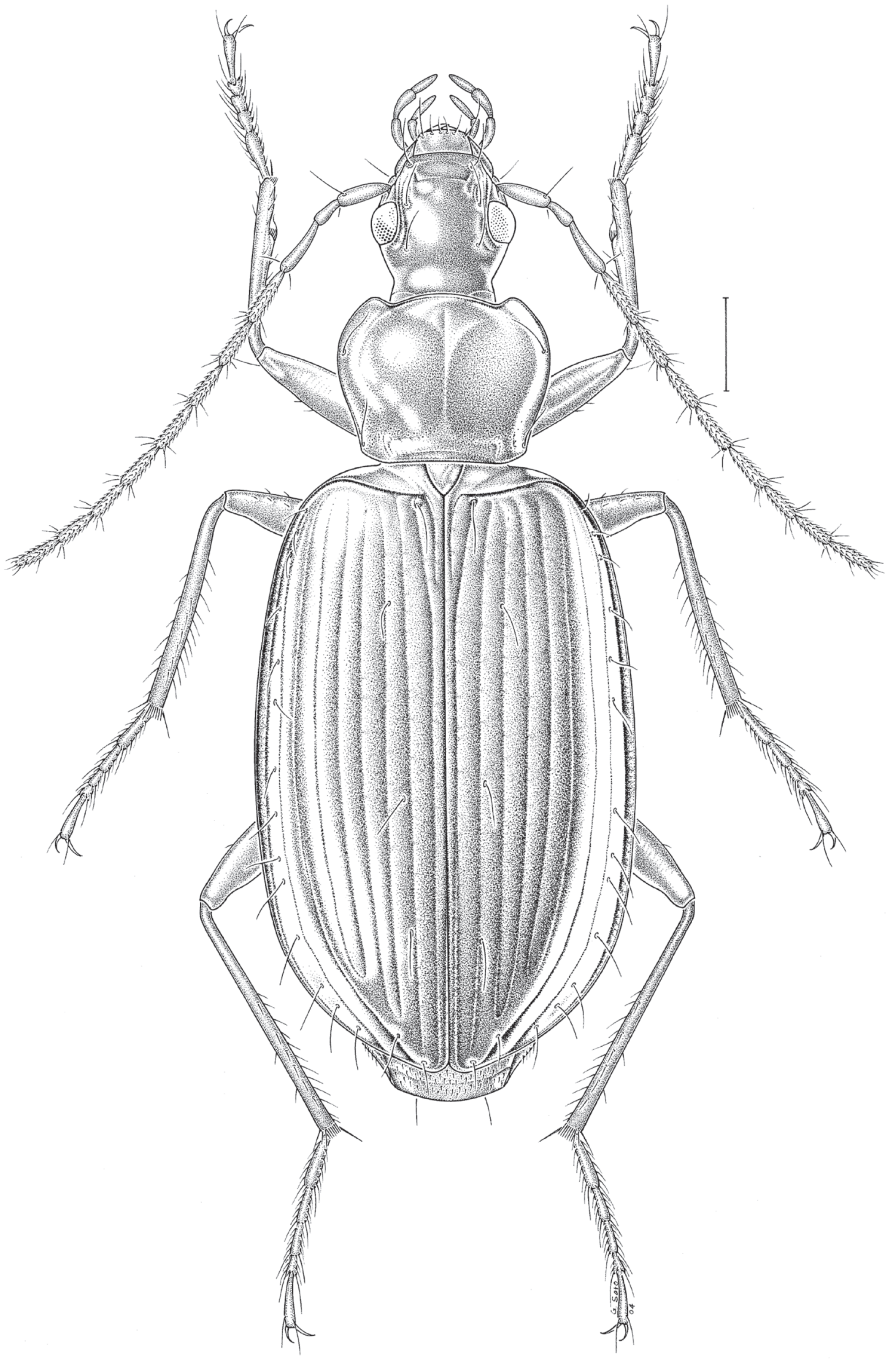
*Platynus daviesi*, habitus (dorsal view). Scale bar = 1 mm.

**Figures 2–3.**
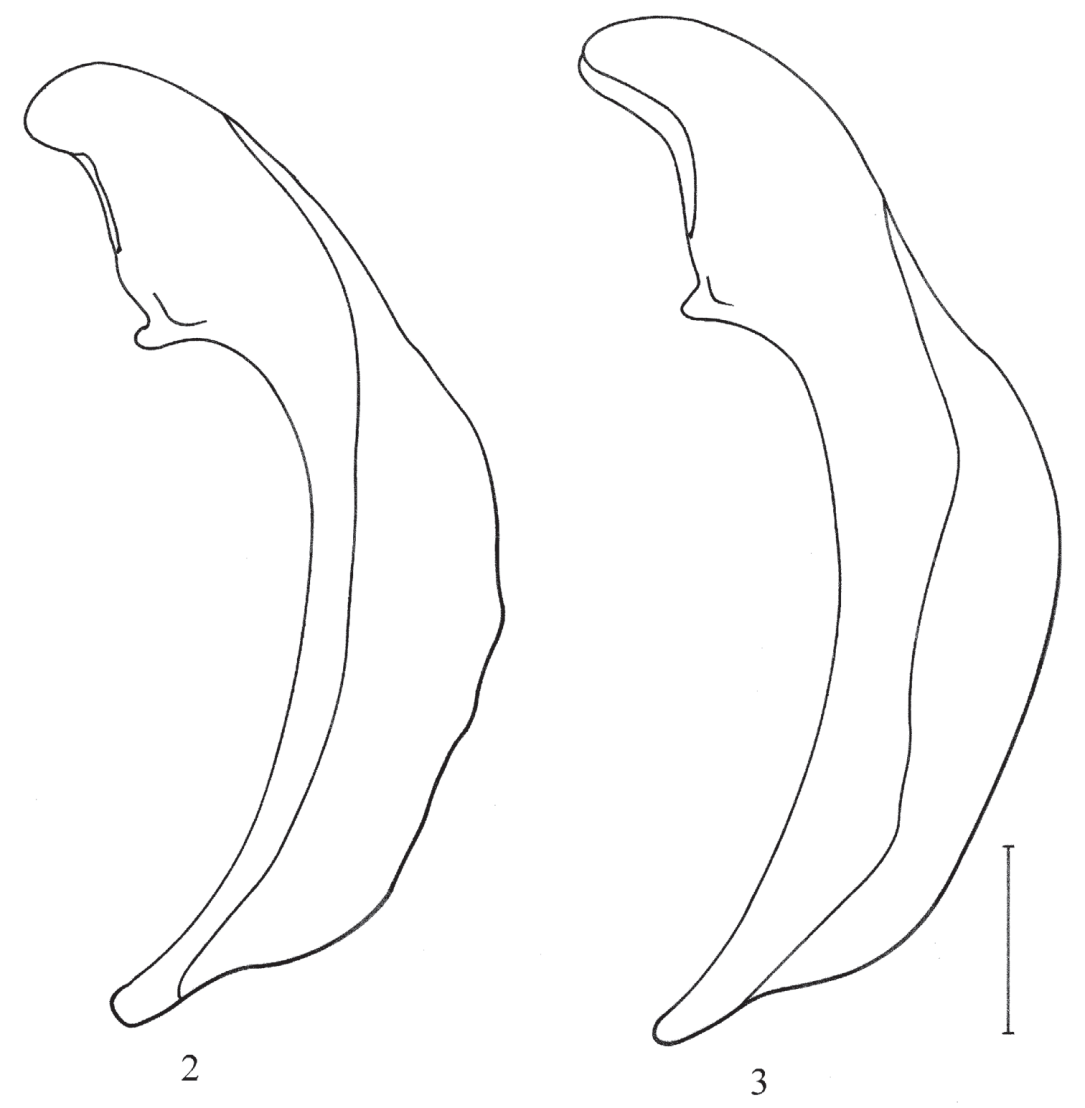
Median lobe of aedeagus (left lateral view) **2** *Platynus daviesi* **3** *Platynus parmarginatus*. Scale bar = 0.3 mm.

Habitus (Fig. 1). *Coloration*. Body dorsally without metallic lustre, brownish red to reddish brown or reddish piceous, with lateral margins of pronotum and elytra paler, yellowish. Antennomeres brownish red to reddish brown, though antennomeres 2 and/or 3 often slightly darker than remaining ones, femora and tibiae also brownish red to reddish brown, though tibiae often slightly paler than femora; tarsomeres paler, yellowish. Frons with two distinct rufous median spots in many specimens. *Microsculpture*. Vertex, less so at centre, with well impressed isodiametric meshes; clypeus with transverse meshes laterally and over anterior half; labrum with well impressed, more or less isodiametric meshes. Pronotum with well impressed moderately transverse meshes on disc, with isodiametric meshes at base between impressions; lateral margins with more or less distinct transverse meshes. Elytra with well impressed, very transverse meshes. *Head*. Eyes moderately protruding. Antennae elongate, antennomere 9 more than four times as long as wide. Mandible not particularly elongate, with retinacular tooth covered by labrum in dorsal view. Anterior edge of mentum tooth not or very slightly emarginate. Submentum with two lateral setae on each side. *Pronotum*. Disc flat. Lateral edge with very shallow sinuation on posterior half. Anterior angle protruding anteriad; posterior angle obtusely rounded. Laterobasal impression deep, rounded, punctate, though in some specimens sparsely so, without convexity. Lateral margin widely reflexed over entire length, punctate toward base, though usually sparsely so; lateral bead indistinct; basal bead indistinct laterally, more or less distinct between impressions. Midlateral and laterobasal setae present; basal seta close to, or even touching, lateral edge. Anterotransverse impression very shallow. *Elytra*. Humerus rounded. Striae moderately finely impressed up to apex, shallowly and sparsely punctate to impunctate; stria 7 usually as impressed as stria 6. Intervals flat; interval 3 with three discal setae on both sides in most specimens (232/285 = 81.5%), rarely with two (3/285 = 1%) on one side or four (39/285 = 13.5%) on one side or four setae (11/285 = 4%) on both sides; anterior seta adjoining stria 3, median and posterior setae close to or adjoining stria 2. Surface around striae 5 and 6 not or only slightly impressed in apical fourth. *Pterothorax*. Metasternum long, its length behind mesocoxa about two times that of longitudinal diameter of mesocoxa. Metepisternum and metasternum impunctate. *Abdomen*. Last visible sternum with two (♂) or four (♀) subapical setae along edge. *Legs*. Mesofemur with three or four ventral setae along posterior edge. Metafemur with zero to two very small dorsoapical setae. Tarsomeres without dorsal keel; metatarsomeres 1 and 2 or 1–3 with shallow lateral furrows but without evident medial furrow; tarsomere 4 symmetric to very slightly asymmetric; tarsomere 5 without setae underneath, though with two to six very small hairs in many specimens. *Genitalia*. Median lobe of aedeagus moderately curved in lateral aspect (Fig. 2); endophallus without sclerified structures.

SBL: 8.7–9.9 mm (mean = 9.2 mm; n = 24).

**Figure 4.**
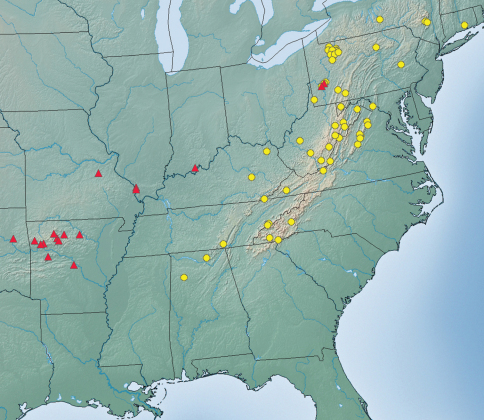
Collection localities for *Platynus daviesi* and *Platynus parmarginatus*. Yellow cercles = *Platynus daviesi*; red triangles = *Platynus parmarginatus*.

#### Etymology.

This species is named for my colleague Anthony Davies, a dedicated research assistant working at Agriculture and Agri-Food Canada, who collected, along with the author, several specimens of the type series.

#### Geographical distribution.

As far as known, this species lives mainly along the Appalachian Mountains, ranging from Connecticut and southern Pennsylvania to northwestern Alabama (Fig. 4).

In addition to records listed in the “Type Material” section, specimens were seen with the following locality labels: “Pen” (1♀, CMNH). “Pa” (1♀, CMNH). “Ohio Pyle,” VII.1905 (1♀, CMNH).

#### Habitat.

Based on information attached to specimen labels, this species is probably an inhabitant of deciduous forests.

#### Dispersal Power.

The wings are fully developed and no doubt functional. Several specimens were collected at ultraviolet light.

#### Comments.

The new species is most similar to *Platynus parmarginatus* Hamilton and *Platynus tenuicollis* (LeConte). Adults of *Platynus parmarginatus* differ from those of *Platynus daviesi* by the following character states: size smaller (SBL: 7.2–8.9 mm; mean= 8.2 mm; n=20) on average; coloration paler on average, more reddish; elytra proportionally shorter and, on average, proportionally narrower (see Table 1); vertex without evident microsculpture meshes; disc of pronotum without or with shallowly impressed, more or less distinct microsculpture microlines; elytral interval 3 with four or five discal setae on both sides in most specimens (77/88 = 88%), rarely with three on one (9/88 = 10%) or both sides (2/88 = 2%); median lobe of aedeagus more curved overall  (Fig. 3). *Platynus parmarginatus* is found mainly west of the Appalachian Mountains (Fig. 4); I have seen specimens of that species from Pennsylvania (Allegheny County), Indiana (Perry County), Illinois (Union County), Missouri (Franklin County), Arkansas (Franklin, Newton, Pope, Stone, and Washington Counties), and Oklahoma (Cherokee County). Both species are sympatric in southwestern Pennsylvania.

**Table 1.** Body proportions for *Platynus daviesi* and *Platynus parmarginatus*

**Species**	**number**	**WH/WP (mean)**	**WP/LP (mean)**	**LE/LP (mean)**	**LE/WE (mean)**
*Platynus daviesi*	24	0.65–0.72 (0.69)	1.19–1.31 (1.25)	3.41–3.73 (3.56)	1.54–1.62 (1.58)
*Platynus parmarginatus*	20	0.65–0.75 (0.70)	1.17–1.34 (1.26)	2.94–3.35 (3.19)	1.45–1.56 (1.50)

While most specimens of *Platynus daviesi* are easy to distinguish from those of *Platynus parmarginatus*, those seen from southwestern Pennsylvania are structurally less distinct. The microsculpture on the vertex and disc of pronotum is less evident on average and the body proportions not so clearly segregated. Nevertheless, all specimens seen could be identified with confidence.

Regarding *Platynus parmarginatus*, Krinsky (1989) pointed out that the median lobes of the aedeagi of his Connecticut specimens did not resemble the median lobe illustrated by Lindroth (1966). In fact, the median lobe illustrated by Krinsky (1989: fig. 1) is that of *Platynus daviesi* while that, extracted from a syntype, illustrated by Lindroth (1966: fig. 323b) belongs to *Platynus parmarginatus*.

Adults of *Platynus tenuicollis* differ from those of *Platynus daviesi* in having the meso- and metatarsomeres 1–3 each with a well-defined dorsal keel and the laterobasal impressions of the pronotum impunctate. *Platynus tenuicollis* is morphologically variable, for example in the elytral microsculpture, and may consist of more than one species.

**Figure 5.**
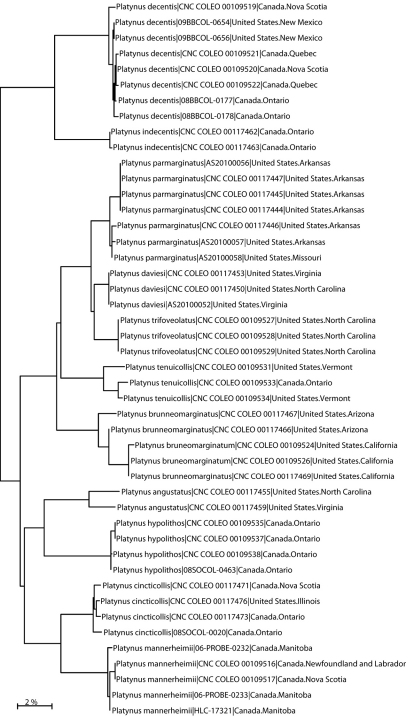
Neighbor-joining tree of genetic distances (Kimura-2-parameter model) of cytochrome c oxidase I (COI) in species of North American *Platynus*. Numbers in parentheses are specimen Sample IDs.

CO1 sequences were analyzed for 46 specimens of *Platynus* representing 11 species (Fig. 5). All barcoded specimens clustered congruently with their respective, morphologically defined species. Mean interspecific divergences ranged from 2.59–15.12% (Table 2). The intraspecific variation ranged from 0.00–1.32% (Table 2), except in *Platynus angustatus* which tabulated at 2.91% suggesting that possibly more than one species are assigned under this name. Indeed, Lindroth (1966: 646) pointed out that the apex of the median lobe in this species varies considerably in length, which is unusual in North American *Platynus* species. Specimens of *Platynus daviesi* were 2.69% divergent from *Platynus parmarginatus*, which is close to the divergence observed between *Platynus trifoveolatus* and *Platynus parmarginatus* (2.98%), two species that have long been separated on the basis of morphological differences. It is interesting to note that *Platynus trifoveolatus*, a morphologically quite isolated species within the eastern North American *Platynus* (see Lindroth 1966: 641), is the species genetically closest to *Platynus daviesi* (2.59%) among the 11 species analyzed (Table 2).

**Table 2.** Percent mitochondrial cytochrome c oxidase I (COI) sequence divergence among species of North American *Platynus* (mean ± standard deviation). Uncorrected average pairwise distances are shown. Cells below diagonal give the mean between-species distances in %. Diagonal (shaded) cells give the mean within-species distances in %. Number of sequences in square brackets after species names.

**SPECIES**	***Platynus angustatus***	***Platynus brunneomarginatus***	***Platynus cincticollis***	***Platynus daviesi***	***Platynus decentis***	***Platynus hypolithos***	***Platynus indecentis***	***Platynus mannerheimii***	***Platynus parmarginatus***	***Platynus tenuicollis***	***Platynus trifoveolatus***
*Platynus angustatus* [2]	2.91±0.89										
*Platynus brunneomarginatus* [5]	9.59±1.69	1.32±0.40									
*Platynus cincticollis* [4]	8.96±1.57	9.54±1.63	0.95±0.39								
*Platynus daviesi* [3]	8.86±1.63	6.84±1.34	10.78±1.72	0.00±0.00							
*Platynus decentis* [8]	13.97±2.19	13.81±1.96	14.11±2.17	13.12±1.89	0.35±0.15						
*Platynus hypolithos* [4]	6.35±1.35	11.74±1.82	9.35±1.54	9.31±1.67	14.23±2.12	0.00±0.00					
*Platynus indecentis* [2]	13.91±2.15	14.47±2.04	13.01±2.06	14.66±2.10	6.46±1.33	13.99±2.15	0.28±0.28				
*Platynus mannerheimii* [5]	11.29±1.85	9.03±1.61	4.97±1.16	11.05±1.78	14.17±2.10	12.26±1.92	13.26±2.00	0.28±0.19			
*Platynus parmarginatus* [7]	8.98±1.62	7.28±1.43	10.93±1.76	2.69±0.84	14.74±1.99	9.45±1.69	15.12±2.09	10.67±1.76	0.41±0.24		
*Platynus tenuicollis* [3]	10.26±1.69	5.87±1.23	8.69±1.61	4.90±1.11	12.82±1.82	11.91±1.88	12.15±1.82	7.79±1.48	4.96±1.11	1.15±0.45	
*Platynus trifoveolatus* [3]	9.37±1.61	7.22±1.44	10.22±1.69	2.59±0.83	14.20±1.95	11.29±1.84	14.67±2.08	11.05±1.79	2.98±0.89	5.72±1.24	0.00±0.00

In order to help with identifying specimens of the new species, a key to all known eastern *Platynus* species is given.

#### Key to species of *Platynus* found east of the Mississippi River

**Table:** 

1	Tarsomere 5 with relatively long setae underneath	2
–	Tarsomere 5 without setae underneath, though with minute hairs in some specimens	4
2 [1]	Legs yellow to reddish yellow. Elytral intervals 3, 5 and 7 each with double rows of setae aligned along striae	*Platynus (Batenus) hypolithos* (Say)
–	At least femora, in most specimens entire legs, reddish brown to black. Elytral intervals 3, 5, and 7 without setae or each with single row of setae not aligned along striae	3
3 [2]	Pronotum narrow, subquadrate (though appearing elongate), with rounded posterior angles; posterolateral setae well removed from angles. Frons with two rufous spots	*Platynus (Batenus) angustatus* Dejean
–	Pronotum wide, transverse (though appearing subquadrate), with denticulate posterior angles; posterolateral setae relatively close to angles. Frons without rufous spots	*Platynus (Platynus) indecentis* Liebherr & Will
4 [1]	Meso- and metatarsomeres 1–3 each with dorsal keel	*Platynus (Platynus) tenuicollis* (LeConte)
–	Meso- and metatarsomeres 1–3 without dorsal keel	5
5 [4]	Elytral microsculpture transverse to striate	6
–	Elytral microsculpture isodiametric or irregularly isodiametric	8
6 [5]	Metasternum short, length behind mesocoxa distinctly shorter than longitudinal diameter of mesocoxa; wings markedly reduced	*Platynus (Platynus) trifoveolatus* Beutenmüller
–	Metasternum long, length behind mesocoxa longer than longitudinal diameter of mesocoxa; wings fully developed	7
7 [6]	Vertex with isodiametric meshes. Pronotal disc with well-impressed microsculpture microlines. Antennomeres 1–3, femora, and tibiae ± reddish brown, tarsomeres paler. Elytral interval 3 with three discal setae at least on one side in most specimens (96% of specimens seen)	*Platynus (Platynus) daviesi* sp. n.
–	Vertex without meshes. Pronotal disc without or with shallowly impressed microlines. Antennomeres 1–3, femora, and tibiae yellowish to reddish, tarsomeres not paler. Elytral interval 3 with four or five discal setae on both side in most specimens (87.5% of specimens seen)	*Platynus (Platynus) parmarginatus* Hamilton
8 [5]	Frons with two rufous spots	9
–	Frons without rufous spots	10
9 [8]	Pronotum without or with very small notch at each posterior angle. Metasternum shorter: length behind mesocoxa shorter than longitudinal diameter of mesocoxa. Antennomeres 1–3 black (except at extremities). Lateral depressions of pronotum not paler than disc	*Platynus (Batenus) mannerheimii* (Dejean)
–	Pronotum with distinct notch at each posterior angle. Metasternum longer: length behind mesocoxa longer than longitudinal diameter of mesocoxa. Antennomeres 1–3 reddish to reddish black. Lateral depressions of pronotum paler than disc	*Platynus (Batenus) cincticollis* (Say)
10 [8]	Mandible markedly elongate, terebral blade narrowly but acutely curved apically, retinacular tooth distinct, not hidden by labrum in dorsal view [*fide*Liebherr (1990)]	*Platynus (Batenus) prognathus* Van Dyke
–	Mandible moderately elongate, terebral blade more widely and less acutely curved apically, retinacular tooth hidden by labrum in dorsal view	11
11 [10]	Pronotum without evident microsculpture. Elytra proportionally shorter (LE/LP = 2.6–2.9; n=12), ± shiny, and ± oval; intervals convex	*Platynus (Platynus) decentis* (Say)
–	Pronotum with distinct microsculpture, particularly toward sides. Elytra proportionally longer (LE/LP = 3.4–3.8; n=12), dull, and ± parallel-sided; intervals flat or only slightly convex	*Platynus (Platynus) opaculus* LeConte

## Supplementary Material

XML Treatment for Platynus daviesi
